# Intervals between response choices on a single-item measure of quality of life

**DOI:** 10.1186/s12955-016-0443-5

**Published:** 2016-03-11

**Authors:** Yves Henchoz, Lionel Meylan, Brigitte Santos-Eggimann, on behalf of the Research Group on the quality of life of older people in cantons of Vaud and Geneva

**Affiliations:** Institute of Social and Preventive Medicine (IUMSP), University of Lausanne Hospital Centre, Route de la Corniche 10, CH-1010 Lausanne, Switzerland

**Keywords:** Quality of life, Single-item, Scale, Intervals, Older people

## Abstract

**Background:**

A single overall rating of quality of life (QoL) is a sensitive method that is often used in population surveys. However, the exact meaning of response choices is unclear. In particular, uneven spacing may affect the way QoL ratings should be analyzed and interpreted. This study aimed to determine the intervals between response choices to a single-item QoL assessment.

**Methods:**

A secondary analysis was conducted on data from the Lc65+ cohort study and two additional, population-based, stratified random samples of older people (*N* = 5,300). Overall QoL was rated as excellent, very good, good, fair or poor. A QoL score (range 0–100) was derived from participants’ answers to a 28-item QoL assessment tool. A transformed QoL score ranging from 1 (poor) to 5 (excellent) was calculated. The same procedure was repeated to compute seven domain-specific QoL subscores (*Feeling of safety*; *Health and mobility*; *Autonomy*; *Close entourage*; *Material resources*; *Esteem and recognition*; *Social and cultural life*).

**Results:**

Mean (95 % confidence intervals) QoL scores were 96.23 (95.81–96.65) for excellent, 93.09 (92.74–93.45) for very good, 81.45 (80.63–82.27) for good, 65.44 (62.67–68.20) for fair and 54.52 (45.31–63.73) for poor overall QoL, corresponding to transformed QoL scores of respectively 5.00, 4.70, 3.58, 2.05, and 1.00. Ordinality of the categories excellent to poor was preserved in all seven QoL subscores.

**Conclusions:**

The excellent-to-poor rating scale provides an ordinal measure of overall QoL. The intervals between response choices are unequal, but an interval scale can be obtained after adequate recoding of excellent, very good, good, fair and poor.

**Electronic supplementary material:**

The online version of this article (doi:10.1186/s12955-016-0443-5) contains supplementary material, which is available to authorized users.

## Introduction

Quality of life (QoL) is a complex and multidimensional construct often assessed using multi-item scales [1, 2]. Such lengthy tools may represent a large burden to participants, particularly in frail older people. Since the number of relevant QoL dimensions and their importance are highly variable between individuals [3], an overall QoL rating reflecting the disparate values and preferences of individuals is preferable in many situations where the purpose is to assess QoL in a broad sense rather than to provide a detailed description of the construct [4–6].

Overall QoL has been measured in older populations using single item questions such as “Would you say your QoL is excellent, very good, good, fair or poor” [5, 7–10], and using other response choices as well [11–13]. Data analysis and interpretation is limited by a lack of clarity around the exact meaning of response choices. The distances between excellent, very good, good, fair and poor were shown to be unequal in the assessment of self-rated health [14–17]. As stated by Perneger et al. [14], the excellent-to-poor self-rated health item should be coded unevenly (excellent = 5; very good = 4.5; good = 3.7; fair = 2; poor = 1). This will not only restore interval properties and hence facilitate any statistical analysis assuming interval rather than ordinal scaled data, but also improve the interpretation of health ratings in community or clinical settings [14]. Nevertheless, uneven spacing still needs to be investigated in the assessment of overall QoL, which encompasses but is not limited to health.

This study aimed to determine the intervals between response choices to a single-item assessment of overall QoL.

## Methods

### Population

A secondary analysis was conducted on data from a previous study on quality of life in older people [18]. Briefly, study participants were community-dwelling adults aged 68 years and older, recruited from the Lausanne cohort 65+ study (Lc65+) and two additional, stratified, random samples selected from population lists in cantons of Vaud and Geneva. This combined dataset was representative of older people in two French-speaking Swiss regions. Persons living in institutions or with advanced dementia were excluded. The protocol was approved by the Ethics Committee of the Faculty of Biology and Medicine of the University of Lausanne.

### Measures

#### Quality of life (QoL)

The questionnaire included a short description of QoL to ensure that participants had a common understanding of the construct:

“There are many factors that determine quality of life. For instance, health but also social network or financial resources can influence it. All factors are not equally important for all of us; for instance, living alone can be problematic for some people but not others. In the following questions, we wish to know what is important for your quality of life, and to what extent some factors are problematic.”

Overall QoL was assessed by a single categorical question: “How do you rate your current quality of life? (excellent, very good, good, fair, poor)”. Corresponding response choices for self-rated health in the French version of the SF-36 were used [19]. In addition, participants were asked to complete a 28-item QoL questionnaire, which was recently developed to reflect the convergence of health, social, cultural and economic factors of older people’s QoL (see Additional file 1) [18]. Previous analyses conducted on an exploratory sample and a validation sample of community-dwelling older people indicated a highly consistent factorial structure comprising seven QoL domains (*Feeling of safety*; *Health and mobility*; *Autonomy*; *Close entourage*; *Material resources*; *Esteem and recognition*; and *Social and cultural life*) [18]. Each one of the 28 QoL items was rated by respondents on its perceived discomfort or dissatisfaction (not at all, a little, a lot), and on its importance to their own QoL (very low, quite low, quite high, very high).

### Demographic and health characteristics

Participants answered questions about living arrangement, education, sex, age, citizenship, medical conditions, depressive symptoms, and disability in basic activities of daily living (BADLs).

### Statistical analysis

A QoL score was calculated by summing answers regarding discomfort to the 28 QoL items (not at all coded as 2, a little coded as 1, a lot coded as 0) weighted (i.e. multiplied) by their respective importance (very low coded as 1, quite low coded as 2, quite high coded as 3, very high coded as 4), dividing by twice the sum of weights, and multiplying by 100 to obtain a percentage score. Outliers (±3 standard deviations from the mean) were excluded in each category of overall QoL. The procedure described above was applied on the constituent items of each QoL domain (see Additional file 1) to obtain its specific subscore. Because the meaning of ‘a little’ may not necessarily stand midway between ‘not at all’ and ‘a lot’, a sensitivity analysis was conducted by coding a little as 0.8 (sensitivity analysis A) and 1.2 (sensitivity analysis B), according to international variations in the meaning of ‘a little’ relative to ‘not at all’ and ‘a lot’, reported elsewhere [15]. As described in previous studies [14, 17], relative intervals between the mean QoL scores of overall ratings excellent, very good, good, fair and poor were used to calculate a transformed QoL score ranging from 1 (poor) to 5 (excellent).

Analyses were conducted using Stata 14.1 software (StataCorp, College Station, TX).

## Results

Of 7,443 eligible participants, 5,300 (71.2 %) completed the questionnaire. After exclusion of participants with missing data (*N* = 610) or outliers (*N* = 76), a total of 4,614 participants were analyzed. Most of them held Swiss citizenship (86.4 %) and were living with others (64.7 %), slightly more than half were women (52.4 %), and one in five reported basic compulsory education (20.0 %), as indicated in Table 1. Mean age was 74.5 years (SD 5.5, range 68–99). A quarter of them reported depressive symptoms (24.1 %), two thirds had at least one medical condition (66.4 %, excluding hypertension and high cholesterol), but disability in BADLs was present in less than one in five participants (18.9 %).

**Table 1:** Characteristics of study participants (*N* = 4,614)

Characteristics	Number	Percent
Sex (women)	2,416	52.4
Age group		
68–75 years	3,015	65.3
76–99 years	1,599	34.7
Swiss citizenship	3,940	86.4
Living arrangement (alone)	1,609	35.3
Education		
Basic compulsory	912	20.0
Apprenticeship	1,692	37.2
Post-compulsory	1,948	42.8
Depressive symptoms	1,091	24.1
Medical conditions		
0	1,536	33.6
1	1,695	37.1
≥ 2	1,338	29.3
Disability in BADLs	860	18.9

*BADLs* basic activities of daily living

**Fig. 1:**
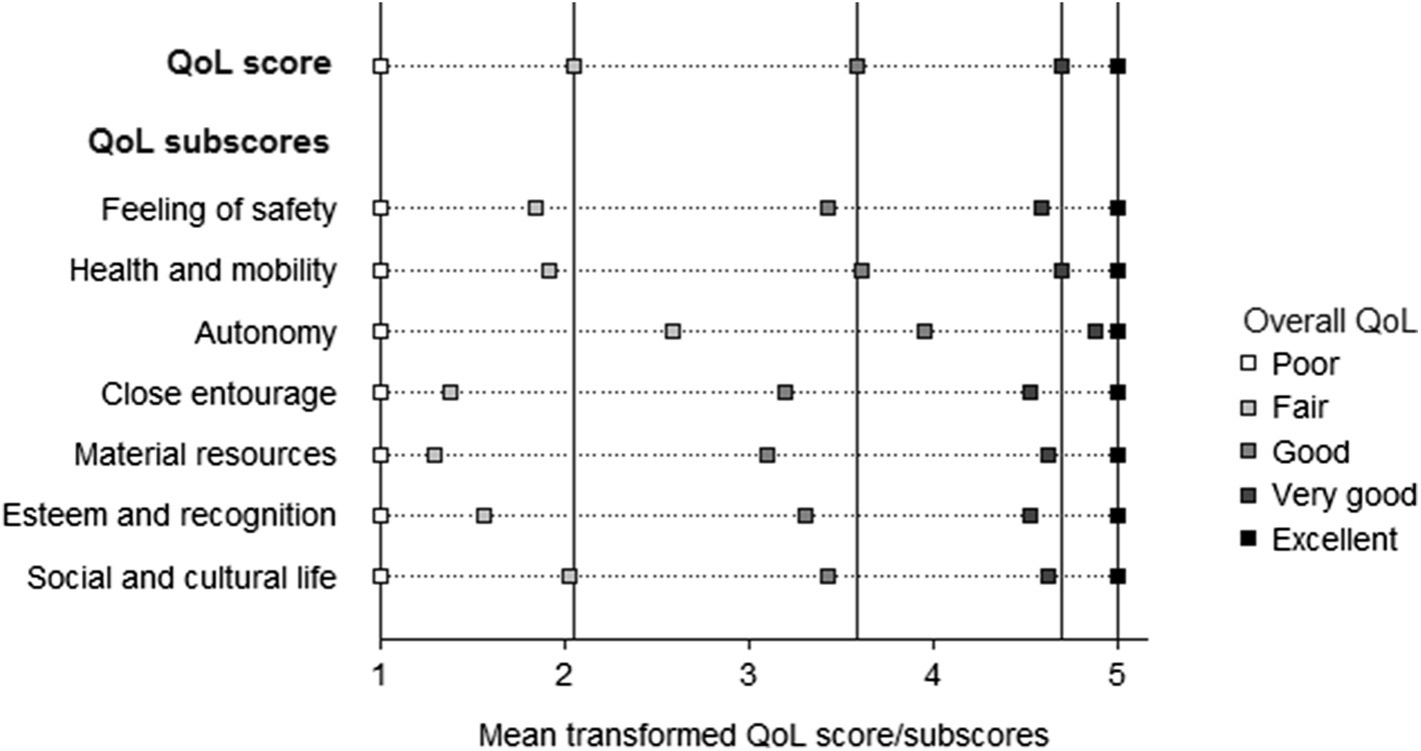
Intervals between poor, fair, good, very good and excellent overall QoL

There was a monotonic increase in the QoL scores across the categories of overall QoL (Table 2). The mean (95 % confidence intervals) QoL scores ranged from 54.52 (45.31–63.73) for poor to 96.23 (95.81–96.65) for excellent. There was only a small overlap in the 95 % confidence intervals of fair and poor QoL scores. Transformed QoL scores were poor = 1.00, fair = 2.05, good = 3.58, very good = 4.70 and excellent = 5.00. Sensitivity analyses A and B confirmed the monotonic increase in the QoL scores across the categories of overall QoL. The position of good slightly above the mid-point of the 1–5 scale was also confirmed, together with the smallest distance observed between excellent and very good and the largest distance observed between good and fair. For all seven QoL domains, mean transformed QoL subscores respected the order of the categories of overall QoL (see Figure 1).

**Table 2:** QoL scores and transformed QoL scores according to overall QoL

	Overall QoL				
	Excellent (*n* = 633)	Very good (*n* = 1,603)	Good (*n* = 2,132)	Fair (*n* = 217)	Poor (*n* = 29)
Main analysis					
QoL score (0–100)	96.23 (95.81–96.65)	93.09 (92.74–93.45)	81.45 (80.63–82.27)	65.44 (62.67–68.20)	54.52 (45.31–63.73)
Transformed QoL score (1–5)	5.00	4.70	3.58	2.05	1.00
Sensitivity analysis A					
QoL score (0–100)	95.60 (95.12–96.08)	91.91 (91.50–92.32)	79.13 (78.27–80.00)	62.00 (59.13–64.87)	51.95 (42.70–61.20)
Transformed QoL score (1–5)	5.00	4.66	3.49	1.92	1.00
Sensitivity analysis B					
QoL score (0–100)	96.85 (96.49–97.21)	94.27 (93.97–94.58)	83.76 (82.98–84.54)	68.87 (66.20–71.55)	57.08 (47.89–66.28)
Transformed QoL score (1–5)	5.00	4.74	3.68	2.19	1.00

Notes: Data are means (95 % confidence intervals); *QoL* quality of life

## Discussion

This population-based study examined the intervals between excellent, very good, good, fair and poor overall QoL among older community dwellings. The results indicated ordinal consistency of the scale, but response choices were unevenly distributed. The smallest gap was observed between very good and excellent, and the largest between fair and good.

Ordinal consistency of the excellent-to-poor overall QoL scale was supported by the monotonic increase in the QoL scores across the categories of overall QoL, with distinct 95 % confidence intervals between excellent, very good, good and fair QoL scores. The little overlap between fair and poor may be explained by a small number of participants in the poor category. Inequality of intervals between excellent, very good, good, fair and poor QoL confirms the findings of previous studies conducted in the field of self-rated health [14–17]. These studies also reported a minimum interval between excellent and very good and a maximum interval between good and fair. Perneger et al. even suggested to add a response label between good and fair [14].

Mean QoL subscores of all seven domains respected the order of the excellent-to-poor overall QoL scale. Hence, at the population level, an overall QoL rating reflects partially all QoL domains. Disparities across domains in the relative distance between adjacent ratings of overall QoL may be linked to variations in the domains on which participants based their overall QoL rating, depending on their level on the excellent-to-poor QoL spectrum. In other words, the domains explaining a fair rather than poor QoL rating appear to differ from those explaining the transition between other adjacent ratings, as has been shown for self-rated health [14].

Hays et al. recently found almost uniform intervals between response choices of the excellent-to-poor health scale using an item response theory (IRT) model [20], suggesting that the intervals may be dependent of the psychometric method used. In the present study, IRT was not used because the purpose was to compare response choices to the excellent-to-poor QoL scale according to a QoL construct that does not involve this single-item scale. The same choice was made by Perneger et al. [14]. Nevertheless, differential item functioning would be a valuable analysis tool in future studies aimed at determining whether intervals between response choices vary according to population characteristics such as age, sex, or education.

In conclusion, the present findings stand for the ordinal consistency of the excellent-to-poor rating scale of overall QoL. Furthermore, an interval scale can reasonably be obtained by recoding poor = 1.00, fair = 2.05, good = 3.58, very good = 4.70 and excellent = 5.00.

## Additional file

Additional file 1: List of 28 quality of life items. (DOCX 11 kb)

## Abbreviations

BADLs: basic activities of daily living
Lc65+: Lausanne cohort 65+
QoL: quality of life
SF-36: 36-item Short Form health survey
