# Supplementary material for: Intervals between response choices on a single-item measure of quality of life
Source: Health Qual Life Outcomes. 2016 Mar 11;14:41. doi: 10.1186/s12955-016-0443-5 (PMC4788954; doi:10.1186/s12955-016-0443-5)
Supplement: Additional file 1: — List of 28 quality of life items. (DOCX 11 kb) [file 12955_2016_443_MOESM1_ESM.docx]

**Additional file 1.** List of 28 quality of life items.

| 1. Financial resources |
| --- |
| 2. Housing comfort |
| 3. Sufficient, good quality food |
| 4. Friendly atmosphere meals |
| 5. Couples' relationships |
| 6. Family relationships |
| 7. Friendship relationships |
| 8. Intergenerational relationships |
| 9. Integration into a group, association or society |
| 10. Self-esteem |
| 11. Being heard and respected |
| 12. Cultural and leisure activities |
| 13. Not being dependent on help in daily life |
| 14. Mobility, being able to travel alone |
| 15. Being able to use public transport alone |
| 16. Being able to travel |
| 17. Physical and mental health |
| 18. Access to health care and prevention |
| 19. Adequate health insurance coverage |
| 20. Safety at home |
| 21. Safety in the street |
| 22. Religion, philosophy or spiritual life |
| 23. Being able to exercise one's creativity, share ideas |
| 24. Being able to decide on issues of daily life |
| 25. Being able to manage money matters alone |
| 26. Being useful to others |
| 27. Being able to express one's opinion, to vote, etc. |
| 28. Being well informed to meet one's needs and decide |
